# Pharmacogenomic biomarkers as source of evidence of the effectiveness and safety of antidepressant therapy

**DOI:** 10.1186/s12888-022-04225-2

**Published:** 2022-08-30

**Authors:** Catarina Correia, Luciano Alcobia, Manuel José Lopes, Ana Margarida Advinha

**Affiliations:** 1 Faculty of Sciences and Technology, University of Algarve, Universidade Do Algarve - Campus de Gambelas, Edifício 8, 8005-139 Faro, Portugal; 2 CHRC – Comprehensive Health Research Centre, University of Evora, Largo do Sr. da Pobreza, 2B, 7000-811 Évora, Portugal; 3 Department of Nursing, São João de Deus School of Nursing, University of Evora, Largo do Sr. da Pobreza, 2B, 7000-811 Evora, Portugal; 4 Department of Medical and Health Sciences, School of Health and Human Development, University of Evora, Rua Romão Ramalho, 59, 7000-671 Evora, Portugal

**Keywords:** Antidepressants, Biomarkers, Depression, Pharmacogenomic, Pharmacotherapy

## Abstract

**Objective:**

The main goal of this work was to identify, describe, characterize, and classify the scientific evidence regarding the use of pharmacogenomic biomarkers in antidepressant treatment.

**Methods:**

The work was developed in two phases: i) a search for pharmacogenomic biomarkers in summaries of antidepressant drugs with marketing authorization in Portugal; and ii) a systematic literature review based on the data obtained in the first phase, with the main objective of finding international literature that could describe and characterize previously reported biomarkers and identify other relevant biomarkers. Finally, the levels of evidence and recommendation grades were classified.

**Results:**

Among the 26 drugs with marketing authorization in Portugal, only 16 had pharmacogenomic information. The most widely studied pharmacogenomic biomarker was CYP2D6. These results were mostly supported by the systematic literature review, which yielded 103 papers, 63 of which were ultimately included in the review. The systematic literature review also revealed the existence of other relevant biomarkers. Most of the included studies show a good level of evidence, which guarantees reliability and good recommendation grades. For the database (built during phase i), the results were informative but resulted in no specific recommendations.

**Conclusions:**

Most pharmacogenomic variants are not studied or acknowledged by genetic tests, and more scientific research is needed to confirm their usefulness. Therefore, only a small number of variants are considered when prescribing antidepressant drugs. In addition, genotyping of patients is not common in clinical practice.

**Supplementary Information:**

The online version contains supplementary material available at 10.1186/s12888-022-04225-2.

## Background

Major depressive disorder (MDD) is a prevalent psychiatric disorder associated with varied prognosis, chronic course, and duration of illness with reduced quality of life. It is characterized by lack of motivation, difficulty in experiencing pleasure, impacts on daily life activities and, in extreme cases, suicide [1, 2].

The World Health Organization declared MDD one of the three leading causes of disability globally, and it is estimated to affect over 260 million people worldwide; additionally, it has a higher prevalence among females than males (5.1% and 3.6%, respectively) [1, 3, 4].

Neurological diseases are difficult to diagnose precisely because of the limited and lack of known biomarkers and the subjectivity of patients’ responses to psychological evaluation questionnaires [2, 5].

A biomarker is a feature that may be reliably examined and assessed as a clinical sign of normal biologic and pathogenic processes, or pharmacologic reactions to a certain therapeutic intervention [2, 4].

Pharmacogenomic biomarkers have recently received attention for their potential to improve antidepressant medication selection and to serve as a prediction tool for increasing treatment response and reducing adverse drug reactions [6].

As a result, there is a pressing need to detect biomarkers capable of predicting treatment response in the future, preferably biomarkers that can distinguish the optimal antidepressant medication for each individual patient. This will result in customized therapy [4].

If a viable biomarker is successfully recognized based on observations indicating a certain molecule may be present or changed exclusively in patients with a given mental condition and not in healthy people, a valid biomarker can be established. However, the basic definition of a psychiatric condition is dependent on subjective and/or behavioral criteria that are clinically defined, which can make establishing whether a person has a specific disorder problematic [7].

The development of such biomarkers will facilitate the evolution of targeted therapy in psychiatry, which will improve treatment effectiveness and can consequently, result in remission as well as on the finding of new targets for the development of innovative antidepressant drugs [4].

In MDD, biomarkers may be used to identify similar groups of patients who will benefit the most from a certain treatment. Biomarkers may also be used to supplement clinical evaluation by emphasizing changes in biomarker levels that occur concurrently with or ahead of changes in clinical symptoms, allowing clinicians to promptly alter medication. The discovery of biomarkers for likely placebo responders might help to cut the size (and hence the expense) of key clinical trials. Finally, biomarkers may one day lead to the selection of more effective and well-tolerated medicines in clinical practice [8].

The main objective of this work was to identify, describe, characterize, and classify the scientific evidence associated with the use of pharmacogenomic biomarkers in the effectiveness and safety of pharmacological treatment of depression.

## Methods

We followed a mixed method, which was divided into two parts: database construction and systematic literature review.

Initially, we constructed a database that included all the information about pharmacogenomic biomarkers found on the summary of product characteristics (SmPC) for the antidepressant drugs with marketing authorization (MA) in Portugal. This was integrated into the Operational Program for Cross-Border Cooperation between Spain and Portugal (*Projeto de Organização e Cooperação Transfronteiriça Espanha Portugal* [POCTEP]) to study the relationship between the pharmacogenomic biomarkers present on the SmPC of the antidepressant drugs, as well as their safety and effectiveness profile. The goals of the database were as follows:

1. To identify antidepressant drugs with MA in Portugal and their SmPC.
2. To identify pharmacogenomic biomarkers of the SmPC of antidepressant drugs with MA in Portugal.
3. To identify, analyze, and classify the information about the pharmacogenomic biomarkers.
4. To compare the information found on the Portuguese SmPC with the available international literature about those biomarkers through a systematic literature review.

### Systematic literature review

After the construction of the database containing the biomarkers identified in the SmPC, we conducted a systematic literature review to search for those pharmacogenomic biomarkers in the international literature, according to Preferred Reporting Items for Systematic reviews and Meta-Analyses (PRISMA) statement (2020) [9].

This systematic literature review aimed to identify and describe evidence regarding effectiveness and safety of using pharmacogenomic biomarkers in antidepressant therapy.

#### Eligibility criteria

The inclusion criteria were as follows: (1) the object of study was one or more antidepressants included in the database previously built; and (2) the presence of pharmacogenomic information. The exclusion criteria were the study was not written in Portuguese or English; or the study did not describe any pharmacogenomic information associated with antidepressants.

#### Information sources

For the systematic literature review, we performed a comprehensive literature search for any type of entries, such as cohort studies, progressive studies and systematic reviews, guidelines, and case reports, that were written in Portuguese or English through October 2020 in PubMed.

#### Search strategy

There were sixteen research questions – one for each antidepressant identified through the construction of the database. The standard question was ((pharmacog*) AND (antidepressant) AND ((pharmacogenomic information obtained through SmPC) OR (pharmacogenomic information))). For example, for the antidepressant clomipramine, the research question was ((pharmacog*) AND (clomipramine) AND ((CYP1A2) OR (CYP3A4) OR (CYP2C19) OR (CYP2D6) OR (CYP*))).

#### Selection process

The study selection process had four steps: 1) screening of the records by title; 2) identification and exclusion of duplicates; 3) screening by abstracts, where we read and analyzed the abstract of each record; and 4) screening through full-text reading, where we read all the studies and excluded those that did not contain any relevant information about antidepressants and/or pharmacogenomics.

The studies obtained from PubMed were extracted through the reference management software Mendeley©, and the data were collected using Microsoft® Office Excel.

The review process was carried out by two of the authors, CC and AMA, and whenever there was doubt regarding the inclusion or exclusion of one of the records/studies, a third author, LA, was consulted to make a final decision.

## Results

According to the Anatomical Therapeutic Chemical Classification System (ATC classification), the group of antidepressants (N06A) has 61 codes, 26 of which have an available SmPC in Portugal. Of those 26 drugs, only 16 had pharmacogenomic information. The pharmacogenomic biomarker that was most widely examined was CYP2D6, an important enzyme for the metabolism of these drugs, which is also highly polymorphic, followed by CYP3A4. CYP3A4 is also highly involved in the metabolism of all drugs, although its polymorphisms are not very relevant. CYP2C19 was also identified in many SmPC, which can be explained by the fact that this enzyme is also very involved in antidepressant metabolism, along with CYP2D6.

Bupropion’s SmPC was the one that covered most information about the presence of pharmacogenomic biomarkers, including six biomarkers: CYP1A2, CYP2A6, CYP3A4, CYP2B6, CYP2C9 and CYP2E1.

All drugs had at least two pharmacogenomic biomarkers, as shown in their SmPC.

As far as scientific evidence is concerned, the results were merely informative, resulting in no specific recommendations. The outcomes were mostly about interactions, which means that the information points out only the possibility of an occurrence of interaction between the drugs associated with those pharmacogenomic biomarkers.

### Systematic literature review

**Fig. 1:**
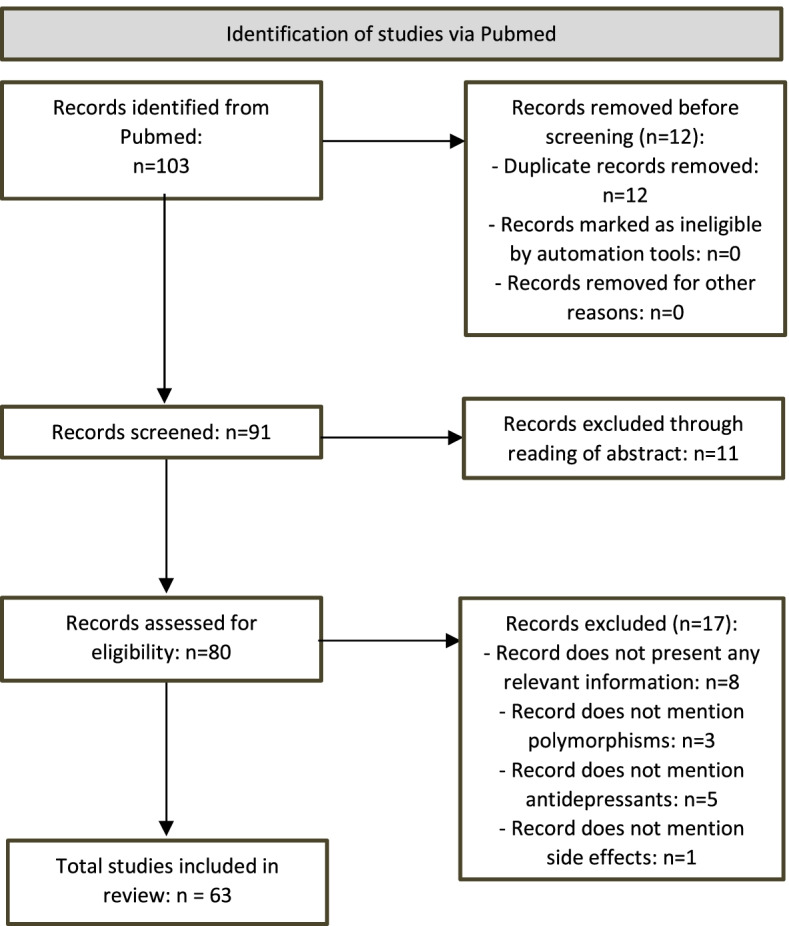
Systematic literature review flowchart

Through the application of the sixteen research questions, we identified 103 records, 12 of which were duplicates. After reading the abstracts, we excluded 11 records. Finally, after fully reading all the records, we excluded 17 records for not having relevant information, resulting in 63 records being included in the systematic literature review (Fig. 1).

Citalopram’s research question was the one that showed the most results, followed by venlafaxine. On the other hand, the research questions for moclobemide, reboxetine, and vortioxetine did not show any results (Table 1).

**Table 1:** Absolute frequency of the records found for each antidepressant drug

Drugs and their respective ATC	Absolute frequency of records
**Clomipramine** (N06AA04)	10
**Maprotiline** (N06AA2)	1
**Fluoxetine** (N06AB03)	11
**Citalopram** (N06AB04)	21
**Sertraline** (N06AB06)	9
**Fluvoxamine** (N06AB08)	3
**Escitalopram** (N06AB10)	10
**Moclobemide** (N06AG02)	0
**Trazodone** (N06AX05)	2
**Mirtazapine** (N06AX11)	5
**Bupropion** (N06AX12)	10
**Venlafaxine** (N06AX16)	18
**Reboxetine** (N06AX18)	0
**Duloxetine** (N06AX21)	2
**Agomelatine** (N06AX22)	1
**Vortioxetine** (N06AX26)	0
**TOTAL**	**103**

The United States of America was the country with the most publications (twenty-two (22)), followed by Canada (eight (8)) and Spain and Germany (six (6) each). Most studies were published between 2011 and 2020. Several types of studies were identified in the systematic literature review, the most frequent being the prospective cohort study (*n* = 10). Most studies included a sample of both sexes (*n* = 38) and/or Caucasian participants (*n* = 26).

The observed outcomes were mostly side effects and lack of effectiveness, as shown in Table 2. In most cases, the most identified biomarkers – CYP2D6 and CYP2C19 – predicted a lack of effectiveness and undesirable effects.

**Table 2:** Number of studies found for each pair of drugs/biomarkers and their outcomes

Pair drug/biomarker		Number of Studies	Outcomes
**Clomipramine**	CYP1A2	0	Not applicable
	CYP3A4	1	Lack of effectiveness
			Undesirable effects
	CYP2C19	6	Lack of effectiveness
			Undesirable effects
	CYP2D6	7	Undesirable effects
	CYP2C9	1	Information not available
**Maprotiline**	CYP1A2	0	Not applicable
	CYP2D6	0	Not applicable
**Fluoxetine**	CYP2D6	5	Lack of effectiveness
			Undesirable effects
			Interactions
	CYP2C9	2	Information not available
	CYP2C19	3	Undesirable effects
**Citalopram**	CYP3A4	0	Not applicable
	CYP2C19	12	Lack of effectiveness
			Undesirable effects
	CYP2D6	4	Undesirable effects
	CYP3A4	1	Information not available
**Sertraline**	CYP3A4	0	Not applicable
	CYP2C19	6	Lack of effectiveness
			Undesirable effects
	CYP2D6	1	Information not available
	CYP2B6	2	Lack of effectiveness
			Undesirable effects
**Fluvoxamine**	CYP2D6	3	Lack of effectiveness
			Undesirable effects
	CYP2C19	1	Information not available
**Escitalopram**	CYP3A4	0	Not applicable
	CYP2C19	6	Lack of effectiveness
			Undesirable effects
	CYP2D6	4	Information not available
**Moclobemide**	CYP2C19	0	Not applicable
	CYP2D6	0	Not applicable
**Trazodone**	CYP3A4	1	Information not available
	CYP2D6	2	Undesirable effects
**Mirtazapine**	CYP1A2	2	Information not available
	CYP3A4	0	Not applicable
	CYP2D6	4	Lack of effectiveness
			Undesirable effects
	CYP2B6	1	Information not available
**Bupropion**	CYP1A2	0	Not applicable
	CYP2A6	0	Not applicable
	CYP3A4	0	Not applicable
	CYP2B6	7	Lack of effectiveness
			Undesirable effects
			Interactions
	CYP2C9	1	Information not available
	CYP2E1	0	Not applicable
	CYP2D6	1	Information not available
	CYP2C19	2	Lack of effectiveness
**Venlafaxine**	CYP3A4	3	Information not available
	CYP2D6	17	Lack of effectiveness
			Undesirable effects
	CYP2C19	9	Interactions
**Reboxetine**	CYP3A4	0	Not applicable
**Duloxetine**	CYP1A2	1	Lack of effectiveness
	CYP2D6	1	Information not available
**Agomelatine**	CYP1A2	2	Undesirable effects
	CYP2C9	1	Information not available
	CYP2C19	0	Not applicable
**Vortioxetine**	CYP3A4	0	Not applicable
	CYP3A5	0	Not applicable
	CYP2C9	0	Not applicable
	CYP2D6	0	Not applicable

To classify the levels of evidence for the studies, as well as the grades of recommendation, we selected the classification created by the University of Oxford, namely, by the Oxford Centre for Evidence Based Medicine (OCEBM) [10]. Most studies included in the systematic literature review were observational, although there were some clinical studies and guidelines. This ensures a good level of evidence, meaning it guarantees reliability and a good recommendation grade. Two authors performed the initial classification and the other two performed a double-blind validation.

## Discussion

Many results were obtained while performing the research. However, not all of them are relevant and/or useful. In this section, we will discuss the most relevant discoveries and provide some recommendations.

The database results were associated with the occurrence of adverse effects and/or interactions between drugs metabolized by polymorphic cytochromes, leading to a variation in the metabolism of the antidepressant and consequent effect. The outcomes of the systematic literature review were the occurrence of adverse effects and/or lack of effectiveness, which means that most results presented a lack of effectiveness when a certain polymorphism was present.

As expected, CYP2D6 was the most frequently identified cytochrome (Table 3), likely because it is strongly involved in the metabolism of antidepressants. CYP2C19 was the second-most frequently identified cytochrome, as it is also involved in the metabolism of most antidepressants.

**Table 3:** Absolute frequency of records found for each pharmacogenomic biomarker

Pharmacogenomic biomarkers	Absolute frequency of records
CYP1A2	4
CYP2B6	10
CYP2C9	6
CYP2C19	31
CYP2D6	41
CYP3A4	6

Usually, the CYP2D6 and CYP2C19 variants, which lead to a faulty cytochrome (and, therefore, lead to a poor metabolizer), also lead to an increase in the drug’s concentration, thereby enhancing the probability of the occurrence of adverse effects and/or toxicity. On the other hand, the variants that lead to an enhanced cytochrome (and, once again, lead to a rapid metabolizer or ultrarapid metabolizer) also lead to faster drug metabolization. This means that the antidepressant may not exert its effect, since it is metabolized more rapidly than expected.

In terms of specific variants, we can conclude that variants *2 of CYP2D6 and *17 of CYP2C19 both give enhanced activity to cytochromes. On the other hand, the variants *3, *4 and *6 of CYP2D6, *2, *3, *4 and *5 of CYP2C19 and *6 of CYP2B6 decrease cytochrome activity.

The drugs clomipramine, fluoxetine, citalopram, fluvoxamine, escitalopram, and duloxetine had biomarkers that were present in the SmPC, in the systematic review, and in the FDA (U. S. Food & Drug Administration) pharmacogenomic biomarkers table [11]. On the other hand, biomarkers for vortioxetine and bupropion were only found in the SmPC and in the FDA pharmacogenomic biomarkers table, but not in the systematic review.

During this research, we encountered some limitations related to the database and limitations related to the systematic literature review. The limitations related to the database included the unavailability of some SmPC data on the Infomed database (the Portuguese drugs database for human use), the probability of non-analyzing some SmPC data due the timeframe in which the work was developed, and the possibility of some actualizations that could not be analyzed for the same reason. The limitations related to the systematic literature review include the difficulty finding records through some research questions. There were also too many results for some drugs, and for others, there were almost none. Due to the heterogeneity of the studies, it was not included a publication bias. Last, we need to take into consideration the possibility of selection bias.

Through the integration and analysis of the information obtained previously through SmPC and systematic literature review, this research made it possible for us to provide recommendations regarding the use of certain antidepressants. For citalopram, we recommend a reduction in the dose for poor metabolizers and an increase of dose for “ultrarapid” metabolizers of CYP2C19, as the variants of this cytochrome have a lot of influence on citalopram’s metabolism and can lead to either toxicity or lack of effectiveness/side effects [12, 13]. For sertraline, we recommend reconsidering switching to another antidepressant that is not metabolized by CYP2C19 in the case of “ultrarapid” metabolizers [14]. For venlafaxine, we recommend the genotyping of CYP2D6 and adjusting the dose if the patient has the phenotype of poor metabolizer, since the standard dose may be toxic to the patient. In the case of a nonresponse to the antidepressant, the dose should not be increased, as this may also lead to toxicity [15–17]. Last, for amitriptyline, we recommend adjusting the dose for either poor or ultrarapid CYP2D6 metabolizers [18].

Generally, we recommend the development of an electronic system integrated into the health system that allows the creation of alerts for pharmacogenomic biomarkers when prescribing and dispensing both antidepressants and other medications that may be influenced by the many existing polymorphisms.

Pharmacogenomic research is an emerging area, with new discoveries being made every day. This constant and rapid evolution creates the need for health care professionals to be constantly updated. Therefore, its implementation is dependent on these health care professionals and patients [19].

On the other hand, we need to take into consideration that only the most prevalent pharmacogenomic variants are genotyped, and thus, new alleles that may contribute to relevant changes in the metabolism of these drugs remain untested. However, these new alleles need to be extensively studied and validated before being included in clinical genotyping [19].

Research in this area will continue to develop, allowing a broader approach to the genotyping of the many variants that influence the metabolism of antidepressants. The scientific evidence, however, remains limited, and a larger investment in this area will be needed, especially in the following fields: studies of new variants in well-studied drugs, studies of relevant variants in non-studied drugs, and studies in different populational groups. There are already some guidelines, such as the Clinical Pharmacogenetics Implementation Consortium (CPIC) [18, 20], but they are nonspecific and do not apply to most antidepressants.

## Conclusions

Major depression is one of the most prevalent mental illnesses and is also difficult to treat. The extension of its effects usually results in incapacitant symptoms (which reduce the quality of life), suicide, lack of productivity and increased health-related costs. Normally, pharmacotherapy is recommended as a first-line treatment, although it is not successful most of the time [21].

Therefore, the use of biomarkers in the management of MDD can be useful, since it would prevent the occurrence of side effects (which can lead to the abandonment of the medication) and thus increase the probability of remission.

The cytochromes CYP2C19 and CYP2D6 are the enzymes with the strongest involvement in antidepressant metabolism. Their polymorphisms are, therefore, more relevant as possible biomarkers.

The variants *2 of CYP2D6 and *17 of CYP2C19 are the most important in regard to the enhanced activity of cytochromes. These variants make the drug metabolism faster than expected, and because of that, the necessary concentration for the drug to do its effect is not reached. Therefore, the antidepressant will not have any effect on the patient (good or bad). An increase in dose is usually related to higher toxicity and should not be considered in cases of nonresponse to the antidepressant. These variants are linked to a rejection of the medication due the lack of effectiveness.

Other variants, such as *2, *3, *4 and *5 of CYP2C19 and *3, *4 and *6 of CYP2D6, lead to a decrease in the activity of these cytochromes, thereby slowing the drug metabolism and leading to its accumulation. This accumulation can lead to a higher risk of side effects that, when intolerable, can lead to the abandonment of the medication.

CYP2B6 is also a notable cytochrome, as it is involved in the metabolism of some antidepressants and has some scientific evidence as a possible biomarker. Variant *6 is the most studied variant, and it gives decreased activity to cytochrome.

The cytochromes CYP3A4, CYP1A2 and CYP2C9 were examined in many of the included studies, but they are not highly relevant biomarkers, as their polymorphisms did not show constant and useful evidence.

## Supplementary Information

**Additional file 1: Supplementary File 1.** Database information by drug/biomarker and recommendations, outcomes, and additional information from SmPC.

**Additional file 2: Supplementary File 2.** Identification of the studies/publications included in the systematic literature review, such as title, authors, country, and reference, generated by the reference management software Mendeley©.

**Additional file 3: Supplementary File 3.** Characterization of the studies/publications according to the type of study, number of samples and their characterization regarding sex, age, and population group.

**Additional file 4: Supplementary File 4.** Tables with the results related to the drugs, namely the information that was found on the SmPC and/or in the systematic literature review, and the comparison with FDA table: • Clomipramine, maprotiline, fluoxetine and citalopram (Table 1 of the Supplementary File 3). • Sertraline, fluvoxamine, escitalopram and moclobemide (Table 2 of the Supplementary File 3). • Trazodone, mirtazapine, bupropion, and venlafaxine (Table 3 of the Supplementary File 3). • Reboxetine, duloxetine, agomelatine and vortioxetine (Table 4 of the Supplementary File 3).

**Additional file 5: Supplementary File 5.** Levels of evidence and GRADE of the studies included in the systematic literature review.

## Data Availability

All data generated or analyzed during this study are included in this published article and in the supplementary files.

## Abbreviations

ATC classification: Anatomical Therapeutic Chemical Classification System
CPIC: Clinical Pharmacogenetics Implementation Consortium
FDA: U.S. Food & Drug Administration
MA: Marketing authorization
MDD: Major depressive disorder
POCTEP: *Projeto de Organização e Cooperação Transfronteiriça Espanha Portugal*
SmPC: Summary of product characteristics
