# Supplementary material for: Pharmacogenomic biomarkers as source of evidence of the effectiveness and safety of antidepressant therapy
Source: BMC Psychiatry. 2022 Aug 30;22:576. doi: 10.1186/s12888-022-04225-2 (PMC9425945; doi:10.1186/s12888-022-04225-2)
Supplement: Supplementary file 1 — Additional file 1: Supplementary File 1. Database information by drug/biomarker and recommendations, outcomes, and additional information from SmPC. [file 12888_2022_4225_MOESM1_ESM.docx]

**SUPPLEMENTARY FILE 1**

| ATC / Drug | CYP1A2 | CYP2A6 | CYP3A4 | CYP3A5 | CYP2B6 | CYP2C9 | CYP2C19 | CYP2D6 | CYP2E1 |
| --- | --- | --- | --- | --- | --- | --- | --- | --- | --- |
| N06AA04  Clomipramine | X |  | X |  |  |  | X | X |  |
| N06AA21  Maprotiline | X |  |  |  |  |  |  | X |  |
| N06AB03  Fluoxetine |  |  |  |  |  |  |  | X |  |
| N06AB04  Citalopram |  |  | X |  |  |  | X | X |  |
| N06AB06  Sertraline |  |  | X |  | X |  | X |  |  |
| N06AB08  Fluvoxamine |  |  |  |  |  |  |  | X |  |
| N06AB10  Escitalopram |  |  | X |  |  |  | X | X |  |
| N06AG02  Moclobemide |  |  |  |  |  |  | X | X |  |
| N06AX05  Trazodone |  |  | X |  |  |  |  |  |  |
| N06AX11  Mirtazapine | X |  | X |  |  |  |  | X |  |
| N06AX12  Bupropion | X | X | X |  | X | X |  |  | X |
| N06AX16  Venlafaxine |  |  | X |  |  |  |  | X |  |
| N06AX18  Reboxetine |  |  | X |  |  |  |  |  |  |
| N06AX21  Duloxetine | X |  |  |  |  |  |  | X |  |
| N06AX22  Agomelatine | X |  |  |  |  | X | X |  |  |
| N06AX26  Vortioxetine |  |  | X | X |  | X |  | X |  |

**Table 1 of Supplementary File 1** Database information by drug/biomarker

**Table 2 of Supplementary File 1** Database information by drug/biomarker recommendations, outcomes, and additional information from SmPC

| ATC / Drug | Biomarker | Recommendation | Outcomes | Additional information |
| --- | --- | --- | --- | --- |
| N06AA04 Clomipramine | CYP1A2 | Informative | Interaction | IBM^[[1]](#footnote-1)^ |
|  | CYP3A4 | Informative | Interaction | IBM |
|  | CYP2C19 | Informative | Interaction | IBM |
|  | CYP2D6 | Informative | Interaction | IBM |
| N06AA21 Maprotiline | CYP1A2 | Informative | Interaction | IBM |
|  | CYP2D6 | Informative | Interaction Undesirable effects | IBM |
| N06AB03 Fluoxetine | CYP2D6 | Informative | Interaction | NR^[[2]](#footnote-2)^; IBM |
| N06AB04 Citalopram | CYP3A4 | Informative | Interaction | IBM |
|  | CYP2C19 | Informative | Interaction | D^[[3]](#footnote-3)^; IBM |
|  | CYP2D6 | Informative | Interaction | IBM |
| N06AB06 Sertraline | CYP3A4 | Informative | Interaction | IBM |
|  | CYP2C19 | Informative | Interaction | IBM |
|  | CYP2B6 | Informative | - | IBM |
| N06AB08 Fluvoxamine | CYP2D6 | Informative | - | IBM |
| N06AB10 Escitalopram | CYP3A4 | Informative | - | IBM |
|  | CYP2C19 | Informative | Interaction | D; IBM |
|  | CYP2D6 | Informative | Interaction | IBM |
| N06AG02 Moclobemide | CYP2C19 | Informative | - | IBM |
|  | CYP2D6 | Informative | - | IBM |
| N06AX05 Trazodone | CYP3A4 | Informative | Interaction Undesirable effects | IBM |
| N06AX11 Mirtazapine | CYP1A2 | Informative | Interaction | - |
|  | CYP3A4 | Informative | Interaction | IBM |
|  | CYP2D6 | Informative | Interaction | IBM |
| N06AX12 Bupropion | CYP1A2 | Informative | - | - |
|  | CYP3A4 | Informative | - | - |
|  | CYP3A5 | Informative | - | - |
|  | CYP2B6 | Informative | Interaction | IBM |
|  | CYP2C9 | Informative | - | - |
|  | CYP2E1 | Informative | - | - |
| N06AX16 Venlafaxine | CYP3A4 | Informative | Interaction | IBM |
|  | CYP2D6 | Informative | Interaction | IBM |
| N06AX18 Reboxetine | CYP3A4 | Informative | Interaction Undesirable effects | NR |
| N06AX21 Duloxetine | CYP1A2 | Informative | Interaction | CI^[[4]](#footnote-4)^; NR; IBM |
|  | CYP2D6 | Informative | Interaction | IBM |
| N06AX21 Agomelatine | CYP1A2 | Informative | Interaction | CI |
|  | CYP2C9 | Informative | - | IBM |
|  | CYP2C19 | Informative | - | - |
| N06AX26 Vortioxetine | CYP3A4 | Informative | Interaction | IBM |
|  | CYP3A5 | Informative | - | - |
|  | CYP2C9 | Informative | Interaction | IBM |
|  | CYP2D6 | Informative | Interaction Undesirable effects | D; IBM |

1. IBM: Information about the biomarker [↑](#footnote-ref-1)
2. NR: Not recommended [↑](#footnote-ref-2)
3. D: Dosage [↑](#footnote-ref-3)
4. CI: Contraindicated [↑](#footnote-ref-4)
