# Supplementary material for: Pharmacogenomic biomarkers as source of evidence of the effectiveness and safety of antidepressant therapy
Source: BMC Psychiatry. 2022 Aug 30;22:576. doi: 10.1186/s12888-022-04225-2 (PMC9425945; doi:10.1186/s12888-022-04225-2)
Supplement: Supplementary file 2 — Additional file 2: Supplementary File 2. Identification of the studies/publications included in the systematic literature review, such as title, authors, country, and reference, generated by the reference management software Mendeley©. [file 12888_2022_4225_MOESM2_ESM.docx]

**SUPPLEMENTARY FILE 2**

Studies identification

| PMID | Title | Authors | Year | Country | Reference |
| --- | --- | --- | --- | --- | --- |
| 16871470 | *Adverse drug reactions following nonresponse in a depressed patient with CYP2D6 deficiency and low CYP 3A4/5 activity* | Stephan PL, Jaquenoud Sirot E, Mueller B, Eap CB, Baumann P. | 2006 | Switzerland | (20) |
| 20531370 | *Association between CYP2C19*17 and metabolism of amitriptyline, citalopram and clomipramine in Dutch hospitalized patients* | de Vos A, van der Weide J, Loovers HM. | 2010 | Netherlands | (21) |
| 27997040 | *Clinical pharmacogenetics implementation consortium guideline (CPIC) for CYP2D6 and CYP2C19 genotypes and dosing of tricyclic antidepressants: 2016 Update* | Hicks JK, Sangkuhl K, Swen JJ, Ellingrod VL, Müller DJ, Shimoda K, Bishop JR, Kharasch ED, Skaar TC, Gaedigk A, Dunnenberger HM, Klein TE, Caudle KE, Stingl JC. | 2016 | United States of America, Canada, Netherlands, Germany, Japan | (16) |
| 19698378 | *Genotypes of cytochrome P450 and clinical response to clomipramine in patients with major depression* | Bouchez J, Dumur V, Lhermitte M, Goudemand M. | 1995 | France | (22) |
| 15168101 | *Impact of polymorphisms of cytochrome-P450 isoenzymes 2C9, 2C19 and 2D6 on plasma concentrations and clinical effects of antidepressants in a naturalistic clinical setting* | Grasmäder K, Verwohlt PL, Rietschel M, Dragicevic A, Müller M, Hiemke C, Freymann N, Zobel A, Maier W, Rao ML. | 2004 | Germany | (23) |
| 28470111 | *Serum clomipramine and desmethylclomipramine levels in a CYP2C19 and CYP2D6 intermediate metabolizer* | Brown JT, Schneiderhan M, Eum S, Bishop JR. | 2017 | United States of America | (24) |
| 32433340 | *Use of antidepressants with pharmacogenetic prescribing guidelines in a 10-year depression cohort of adult primary care patients* | Jessel CD, Mostafa S, Potiriadis M, Everall IP, Gunn JM, Bousman CA. | 2020 | Canada, Australia | (25) |
| 24663076 | *Effect of CYP2D6, CYP2C9 and ABCB1 genotypes on fluoxetine plasma concentrations and clinical improvement in children and adolescent patients* | Gassó P, Rodríguez N, Mas S, Pagerols M, Blázquez A, Plana MT, Torra M, Lázaro L, Lafuente A. | 2014 | Spain | (26) |
| 22791347 | *Fluoxetine pharmacogenetics in child and adult populations* | Blazquez A, Mas S, Plana MT, Lafuente A, Lázaro L. | 2012 | Spain | (27) |
| 23799451 | *Novel CYP2D6 and CYP2C19 variants identified in a patient with adverse reactions towards venlafaxine monotherapy and dual therapy with nortriptyline and fluoxetine* | Chua EW, Foulds J, Miller AL, Kennedy MA. | 2013 | Malaysia, New Zealand | (28) |
| 27289413 | *Pharmacokinetic Pharmacogenetic Prescribing Guidelines for Antidepressants: A Template for Psychiatric Precision Medicine* | Nassan M, Nicholson WT, Elliott MA, Rohrer Vitek CR, Black JL, Frye MA. | 2016 | United States of America | (29) |
| 31664715 | *A Call for Clear and Consistent Communications Regarding the Role of Pharmacogenetics in Antidepressant Pharmacotherapy* | Hicks JK, Bishop JR, Gammal RS, Sangkuhl K, Bousman CA, Leeder JS, Llerena A, Mueller DJ, Ramsey LB, Scott SA, Skaar TC, Caudle KE, Klein TE, Gaedigk A. | 2019 | United States of America | (10) |
| 31112844 | *Antidepressant pharmacogenetics in children and young adults: A systematic review* | Maruf AA, Greenslade A, Arnold PD, Bousman C. | 2019 | Canada | (30) |
| 25974703 | *Clinical Pharmacogenetics Implementation Consortium (CPIC) Guideline for CYP2D6 and CYP2C19 Genotypes and Dosing of Selective Serotonin Reuptake Inhibitors* | Hicks JK, Bishop JR, Sangkuhl K, Müller DJ, Ji Y, Leckband SG, Leeder JS, Graham RL, Chiulli DL, LLerena A, Skaar TC, Scott SA, Stingl JC, Klein TE, Caudle KE, Gaedigk A | - | United States of America | (18) |
| 30173302 | *Completed suicides of citalopram users-the role of CYP genotypes and adverse drug interactions* | Rahikainen AL, Vauhkonen P, Pett H, Palo JU, Haukka J, Ojanperä I, Niemi M, Sajantila A | 2018 | Finland | (31) |
| 21192344 | *CYP2C19 variation and citalopram response* | Mrazek DA, Biernacka JM, O'Kane DJ, Black JL, Cunningham JM, Drews MS, Snyder KA, Stevens SR, Rush AJ, Weinshilboum RM. | 2011 | United States of America | (32) |
| 27016952 | *Effect of CYP2D6 genetic polymorphism on the metabolism of citalopram in vitro* | Hu XX, Yuan LJ, Fang P, Mao YH, Zhan YY, Li XY, Dai DP, Cai JP, Hu GX. | 2016 | China | (33) |
| 24257813 | *Genetic differences in cytochrome P450 enzymes and antidepressant treatment response* | Hodgson K, Tansey K, Dernovsek MZ, Hauser J, Henigsberg N, Maier W, Mors O, Placentino A, Rietschel M, Souery D, Smith R, Craig IW, Farmer AE, Aitchison KJ, Belsey S, Davis OS, Uher R, McGuffin P. | 2013 | United Kingdom | (34) |
| 30837874 | *Influence of CYP2C19 Metabolizer Status on Escitalopram/Citalopram Tolerability and Response in Youth With Anxiety and Depressive Disorders* | Aldrich SL, Poweleit EA, Prows CA, Martin LJ, Strawn JR, Ramsey LB. | 2019 | United States of America | (35) |
| 29712478 | *Mindful Pharmacogenetics: Drug Dosing for Mental Health* | Stingl JC | 2018 | Germany | (11) |
| 12975335 | *Pharmacokinetics of citalopram in relation to genetic polymorphism of CYP2C19* | Yu BN, Chen GL, He N, Ouyang DS, Chen XP, Liu ZQ, Zhou HH. | 2003 | China | (36) |
| 16855453 | *Phenotype-genotype relationship and clinical effects of citalopram in Chinese patients* | Yin OQ, Wing YK, Cheung Y, Wang ZJ, Lam SL, Chiu HF, Chow MS. | 2006 | China | (37) |
| 29136336 | *Effect of Polymorphisms on the Pharmacokinetics, Pharmacodynamics and Safety of Sertraline in Healthy Volunteers* | Saiz-Rodríguez M, Belmonte C, Román M, Ochoa D, Koller D, Talegón M, Ovejero-Benito MC, López-Rodríguez R, Cabaleiro T, Abad-Santos F. | 2017 | Spain | (12) |
| 31649299 | *Impact of CYP2C19 genotype on sertraline exposure in 1200 Scandinavian patients* | Bråten LS, Haslemo T, Jukic MM, Ingelman-Sundberg M, Molden E, Kringen MK. | 2019 | Norway | (38) |
| 26830411 | *Influence of CYP2B6 and CYP2C19 polymorphisms on sertraline metabolism in major depression patients* | Yuce-Artun N, Baskak B, Ozel-Kizil ET, Ozdemir H, Uckun Z, Devrimci-Ozguven H, Suzen HS. | 2016 | Turkey | (39) |
| 11452243 | *Pharmacokinetics of sertraline in relation to genetic polymorphism of CYP2C19* | Wang JH, Liu ZQ, Wang W, Chen XP, Shu Y, He N, Zhou HH. | 2001 | China | (40) |
| 20547595 | *CYP2D6 genotype and smoking influence fluvoxamine steady-state concentration in Japanese psychiatric patients: lessons for genotype-phenotype association study design in translational pharmacogenetics* | Suzuki Y, Sugai T, Fukui N, Watanabe J, Ono S, Inoue Y, Ozdemir V, Someya T. | 2011 | Japan, Canada | (41) |
| 29988737 | *Effects of CYP2D6 genetic polymorphisms on the efficacy and safety of fluvoxamine in patients with depressive disorder and comorbid alcohol use disorder* | Zastrozhin MS, Grishina EA, Denisenko NP, Skryabin VY, Markov DD, Savchenko LM, Bryun EA, Sychev DA. | 2018 | Russia | (42) |
| 25200585 | *Clinical applications of CYP genotyping in psychiatry* | Spina E, de Leon J. | 2014 | Italy, Spain, United States of America | (43) |
| 21926427 | *CYP2C19 genotype predicts steady state escitalopram concentration in GENDEP* | Huezo-Diaz P, Perroud N, Spencer EP, Smith R, Sim S, Virding S, Uher R, Gunasinghe C, Gray J, Campbell D, Hauser J, Maier W, Marusic A, Rietschel M, Perez J, Giovannini C, Mors O, Mendlewicz J, McGuffin P, Farmer AE, Ingelman-Sundberg M, Craig IW, Aitchison KJ | 2012 | United States of America | (44) |
| 24302953 | *CYP2D6 P34S Polymorphism and Outcomes of Escitalopram Treatment in Koreans with Major Depression* | Han KM, Chang HS, Choi IK, Ham BJ, Lee MS. | 2013 | South Korea | (45) |
| 20350136 | *Genetic polymorphisms of cytochrome P450 enzymes influence metabolism of the antidepressant escitalopram and treatment response* | Tsai MH, Lin KM, Hsiao MC, Shen WW, Lu ML, Tang HS, Fang CK, Wu CS, Lu SC, Liu SC, Chen CY, Liu YL. | 2010 | Taiwan | (46) |
| 29325448 | *Impact of CYP2C19 Genotype on Escitalopram Exposure and Therapeutic Failure: A Retrospective Study Based on 2,087 Patients* | Jukić MM, Haslemo T, Molden E, Ingelman-Sundberg M. | 2017 | Norway, Serbia | (47) |
| 24014145 | *Pharmacogenetic polymorphisms and response to escitalopram and venlafaxine over 8 weeks in major depression* | Ng C, Sarris J, Singh A, Bousman C, Byron K, Peh LH, Smith DJ, Tan CH, Schweitzer I. | 2013 | Australia, Singapore | (48) |
| 29061081 | *Pharmacogenetics of trazodone in healthy volunteers: association with pharmacokinetics, pharmacodynamics and safety* | Saiz-Rodríguez M, Belmonte C, Derqui-Fernández N, Cabaleiro T, Román M, Ochoa D, Talegón M, Ovejero-Benito MC, Abad-Santos F. | 2017 | Spain | (49) |
| 9335086 | *Relationship between the CYP2D6 genotype and the steady-state plasma concentrations of trazodone and its active metabolite m-chlorophenylpiperazine* | Mihara K, Otani K, Suzuki A, Yasui N, Nakano H, Meng X, Ohkubo T, Nagasaki T, Kaneko S, Tsuchida S, Sugawara K, Gonzalez FJ. | 1997 | Japan | (50) |
| 31100205 | *Effects of CYP2D6 activity on the efficacy and safety of mirtazapine in patients with depressive disorders and comorbid alcohol use disorder* | Zastrozhin MS, Skryabin VY, Smirnov VV, Grishina EA, Ryzhikova KA, Chumakov EM, Bryun EA, Sychev DA. | 2019 | Russia | (51) |
| 26595747 | *Factors Affecting Steady-state Plasma Concentrations of Enantiomeric Mirtazapine and its Desmethylated Metabolites in Japanese Psychiatric Patients* | Hayashi Y, Watanabe T, Aoki A, Ishiguro S, Ueda M, Akiyama K, Kato K, Inoue Y, Tsuchimine S, Yasui-Furukori N, Shimoda K. | 2015 | United States of America | (52) |
| 22926595 | *Multicenter study on the clinical effectiveness, pharmacokinetics, and pharmacogenetics of mirtazapine in depression* | Jaquenoud Sirot E, Harenberg S, Vandel P, Lima CA, Perrenoud P, Kemmerling K, Zullino DF, Hilleret H, Crettol S, Jonzier-Perey M, Golay KP, Brocard M, Eap CB, Baumann P. | 2012 | Switzerland | (53) |
| 14514498 | *Pharmacogenetics of antidepressant medication intolerance* | Murphy GM Jr, Kremer C, Rodrigues HE, Schatzberg AF. | 2003 | United States of America | (54) |
| 14515060 | *Bupropion and 4-OH-bupropion pharmacokinetics in relation to genetic polymorphisms in CYP2B6* | Julia Kirchheinera, Christian Kleina, Ingolf Meinekeb, Johanna Sassea, Ulrich M. Zangerc, Thomas E. Mürdterc, Ivar Rootsa and Jürgen Brockmöllerb | 2003 | Germany | (55) |
| 26608082 | *Developmental Expression of CYP2B6: A Comprehensive Analysis of mRNA Expression, Protein Content and Bupropion Hydroxylase Activity and the Impact of Genetic Variation* | Robin E. Pearce, Roger Gaedigk, Greyson P. Twist, Hongying Dai, Amanda K. Riffel, J. Steven Leeder e Andrea Gaedigk | 2015 | United States of America | (56) |
| 23344581 | *Influence of CYP2B6 genetic variants on plasma and urine concentrations of bupropion and metabolites at steady state* | Neal L. Benowitz, Andy Z. X. Zhu, Rachel F. Tyndale, Delia Dempsey Peyton Jacob III | 2013 | Canada, United States of America | (57) |
| 23238783 | *The influence of sex, ethnicity, and CYP2B6 genotype on bupropion metabolism as an index of hepatic CYP2B6 activity in humans* | Katarina Ilic, Roy L. Hawke, Ranjit K. Thirumaran, Erin G. Schuetz, J. Heyward Hull, Angela D. M. Kashuba, Paul W. Stewart, Celeste M. Lindley, Mei-Ling Chen | 2013 | Canada, United States of America | (58) |
| 15083067 | *Pharmacogenetic determinants of interindividual variability in bupropion hydroxylation by cytochrome P450 2B6 in human liver microsomes* | Leah M. Hesse, Ping He, Soundararajan Krishnaswamy, Qin Hao, Kirk Hogan, Lisa L. von Moltke, David J. Greenblatt e Michael H. Court | 2004 | United States of America | (59) |
| 28685396 | *Pharmacokinetics and Pharmacogenomics of Bupropion in Three Different Formulations with Different Release Kinetics in Healthy Human Volunteers* | Connarn JN, Flowers S, Kelly M, Luo R, Ward KM, Harrington G, Moncion I, Kamali M, McInnis M, Feng MR, Ellingrod V, Babiskin A, Zhang X, Sun D. | 2017 | United States of America | (60) |
| 32475982 | *Subtherapeutic bupropion and hydroxybupropion serum concentrations in a patient with CYP2C19*1/*17 genotype suggesting a rapid metabolizer status* | Arnim Johannes Gaebler, Katharina Luise Schneider, Julia Carolin Stingl, Michael Paulzen | 2020 | Germany | (61) |
| 16642541 | *CYP2D6 genotype and venlafaxine-XR concentrations in depressed elderly* | Whyte EM, Romkes M, Mulsant BH, Kirshne MA, Begley AE, Reynolds CF 3rd, Pollock BG. | 2006 | Canada | (62) |
| 16958828 | *CYP2D6 polymorphism and clinical effect of the antidepressant venlafaxine* | Shams ME, Arneth B, Hiemke C, Dragicevic A, Müller MJ, Kaiser R, Lackner K, Härtter S. | 2006 | Germany | (14) |
| 17803873 | *Cytochrome P450 2D6 genotype variation and venlafaxine dosage* | McAlpine DE, O'Kane DJ, Black JL, Mrazek DA. | 2007 | United States of America | (63) |
| 19822698 | *Depressive effect of an antidepressant: therapeutic failure of venlafaxine in a case lacking CYP2D6 activity* | Wijnen PA, Limantoro I, Drent M, Bekers O, Kuijpers PM, Koek GH. | 2009 | Netherlands | (64) |
| 26406933 | *Effect of CYP2D6 variants on venlafaxine metabolism in vitro* | Zhan YY, Liang BQ, Wang H, Wang ZH, Weng QH, Dai DP, Cai JP, Hu GX. | 2015 | China | (65) |
| 21099743 | *Effect of cytochrome P450 enzyme polymorphisms on pharmacokinetics of venlafaxine* | McAlpine DE, Biernacka JM, Mrazek DA, O'Kane DJ, Stevens SR, Langman LJ, Courson VL, Bhagia J, Moyer TP. | 2011 | United States of America | (66) |
| 24941211 | *High-dose venlafaxine treatment in a depressed patient with a genetic CYP2D6 deficiency* | Haller-Gloor F, Eap CB, Turgeon J, Baumann P. | 2004 | Canada | (15) |
| 29327975 | *Impact of CYP2D6 on venlafaxine metabolism in Trinidadian patients with major depressive disorder* | Montané Jaime LK, Paul J, Lalla A, Legall G, Gaedigk A. | 2017 | United States of America | (67) |
| 25245581 | *Influence of CYP2D6 and CYP2C19 genotypes on venlafaxine metabolic ratios and stereoselective metabolism in forensic autopsy cases* | Karlsson L, Zackrisson AL, Josefsson M, Carlsson B, Green H, Kugelberg FC. | 2014 | Sweden | (68) |
| 30578947 | *Pharmacokinetic-Pharmacodynamic interaction associated with venlafaxine-XR remission in patients with major depressive disorder with history of citalopram / escitalopram treatment failure* | Ahmed AT, Biernacka JM, Jenkins G, Rush AJ, Shinozaki G, Veldic M, Kung S, Bobo WV, Hall-Flavin DK, Weinshilboum RM, Wang L, Frye MA. | 2018 | United States of America | (69) |
| 28480819 | *Should a routine genotyping of CYP2D6 and CYP2C19 genetic polymorphisms be recommended to predict venlafaxine efficacy in depressed patients treated in psychiatric settings?* | Taranu A, Colle R, Gressier F, El Asmar K, Becquemont L, Corruble E, Verstuyft C. | 2017 | France | (70) |
| 30312494 | *Significantly lower CYP2D6 metabolism measured as the O/N-desmethylvenlafaxine metabolic ratio in carriers of CYP2D6*41 versus CYP2D6*9 or CYP2D6*10: a study on therapeutic drug monitoring data from 1003 genotyped Scandinavian patients* | Haslemo T, Eliasson E, Jukić MM, Ingelman-Sundberg M, Molden E. | - | Norway | (71) |
| 31368838 | *Venlafaxine pharmacogenetics: a comprehensive review* | Suwała J, Machowska M, Wiela-Hojeńska A. | 2019 | Poland | (13) |
| 28520361 | *Venlafaxine Therapy and CYP2D6 Genotype* | Dean L. | 2015, atualizado em 2020 | United States of America | (72) |
| 21366359 | *Duloxetine: clinical pharmacokinetics and drug interactions* | Knadler MP, Lobo E, Chappell J, Bergstrom R. | 2011 | United States of America | (73) |
| 30789308 | *Polymorphisms in CYP1A2, CYP2C9 and ABCB1 affect agomelatine pharmacokinetics* | Miriam Saiz-Rodríguez, Dolores Ochoa, Carmen Belmonte, Manuel Román, Danilo Vieira de Lara, Pablo Zubiaur, Dora Koller, Gina Mejía Francisco Abad-Santos | 2019 | Spain | (74) |
