# Supplementary material for: Pharmacogenomic biomarkers as source of evidence of the effectiveness and safety of antidepressant therapy
Source: BMC Psychiatry. 2022 Aug 30;22:576. doi: 10.1186/s12888-022-04225-2 (PMC9425945; doi:10.1186/s12888-022-04225-2)
Supplement: Supplementary file 3 — Additional file 3: Supplementary File 3. Characterization of the studies/publications according to the type of study, number of samples and their characterization regarding sex, age, and population group. [file 12888_2022_4225_MOESM3_ESM.docx]

**SUPPLEMENTARY FILE 3**

Studies characterization

| Study/Publication Identification | Type of Study/Publication | Unit of Analysis | | | |
| --- | --- | --- | --- | --- | --- |
| PMID |  | Sample | Description | | |
|  |  |  | Sex | Age | Population Group |
| 16871470 | Case report | 1 patient with depression | Male | 47 | Caucasian |
| 20531370 | Retrospective cohort study | 678 patients with depression | Both (♀= 64%) | 18-65+ | N/A |
| 27997040 | Guideline | N/A | N/A | N/A | N/A |
| 19698378 | Prospective cohort study | 19 patients with depression | Both | 19-62 | Caucasian |
| 15168101 | Exploratory study | 136 patients with depression | Both (♀= 56.5%) | 39-63 | Caucasian |
| 28470111 | Case report | 1 patient with depression | Male | 29 | Caucasian |
| 32433340 | Prospective cohort study | 789 patients with depression | Both | 37-61 | Caucasian |
| 24663076 | Prospective cohort study | 83 patients with depression | Both (♀= 68.7%) | 10-17 | Caucasian |
| 22791347 | Scoping review | N/A | N/A | N/A | N/A |
| 23799451 | Case report | 1 patient with depression | Female | 24 | Indian |
| 27289413 | Guideline | N/A | N/A | N/A | N/A |
| 31664715 | Comment | N/A | N/A | N/A | N/A |
| 31112844 | Systematic literature review | 24 studies | N/A | <25 | N/A |
| 25974703 | Guideline | N/A | N/A | N/A | N/A |
| 30173302 | Case-control study | 349 cases of suicide  855 healthy individuals | Both (♀=69.9%) | 30-82 | Caucasian |
| 21192344 | Prospective cohort study | 1503 patients with depression | Both | 18-75 | White, non-Hispanic;  Hispanic white;  Black |
| 27016952 | In vitro preclinical study | Wild type allele of CYP2C19 and its 24 variants | N/A | N/A | N/A |
| 24257813 | Partially randomized, open-label, multicenter study | 868 patients with depression | Both (♀=63%) | 19-72 | Caucasian |
| 30837874 | Retrospective cohort study | 263 children with depression | Both (♀=65%) | <19 | N/A |
| 29712478 | Editorial | N/A | N/A | N/A | N/A |
| 12975335 | Randomized controlled clinical trial | 13 healthy individuals | Male | 20-22 | Chinese |
| 16855453 | Prospective cohort study | 53 patients with depression | Both (♀=67.9%) | 29-79 | Chinese |
| 29136336 | Randomized, open-label, crossover, two-period, two-sequence, single-center clinical trials | 48 healthy individuals | Both | 18-55 | Caucasian |
| 31649299 | Retrospective cohort study | 1202 patients with depression | Both (♀=21.8%) | 18-69 | Scandinavian (mostly caucasian) |
| 26830411 | Cross-sectional study | 50 patients with depression | Both (♀=66) | 18-65 | Turkish |
| 11452243 | Randomized controlled clinical trial | 77 healthy individuals | Male | 19-22 | Chinese |
| 20547595 | Cross-sectional study | 87 patients with depression | Both (♀=34.5%) | 25-48 | Japonese |
| 29988737 | Prospective cohort study | 45 patients with depression | Male | 26-46 | Russian |
| 25200585 | Critical review | N/A | N/A | N/A | N/A |
| 21926427 | Prospective cohort study | 196 patients with depression | Both (♀=66.8%) | 18-66 | Caucasian |
| 24302953 | Non-randomized clinical trial | 94 patients with depression | Both (♀=86.2%) | 28-63 | Korean |
| 20350136 | Observational case-only design study | 100 patients with depression | Both | >18 | Chinese |
| 29325448 | Retrospective cohort study | 2087 patients with depression | Both (♀=63.3%) | N/A | Caucasian |
| 24014145 | Non-randomized clinical trial | 62 patients taking escitalopram; 44 taking venlafaxine | Both (♀=60.4%) | 31-57 | Caucasian and chinese |
| 29061081 | Randomized, open-label, crossover, two-period, two-sequence, single-center, blinded clinical trial | 36 healthy individuals | Both (♀=50%) | 21-29 | Mostly caucasian |
| 9335086 | Cross-sectional study | 54 patients with depression | Both (♀=61.1%) | 30-60 | Japanese |
| 31100205 | Prospective cohort study | 109 patients with depression | Male | 26-46 | Caucasian |
| 26595747 | Cross-sectional study | 77 patients with depression | Both (♀=57.1%) | 17-88 | Japanese |
| 22926595 | Multicenter non-randomized clinical trial | 45 patients with depression | Both (♀=71.1%) | 19-79 | Caucasian |
| 14514498 | Double-blind, randomized clinical trial | 124 patients taking mirtazapine; 122 taking paroxetine | Both (♀=51.9%) | 65-77 | N/A |
| 14515060 | Randomized controlled clinical trial | 121 healthy individuals | Male | 21-31 | Caucasian |
| 26608082 | In vitro preclinical study | 201 pre and postnatal liver samples | N/A | N/A | N/A |
| 23344581 | Randomized controlled clinical trial | 42 healthy individuals | Both (♀=38%) | 10-60 | Hispanic, Non-Hispanic, African American and Asian |
| 23238783 | Non-randomized clinical trial | 4 healthy individuals cohorts | Both | 18-49 | Caucasian-American; Hispanic and African American |
| 15083067 | In vitro preclinical study | 54 liver samples | Both (♀=29.6%) | 2-75 | Caucasian, African American, Hispanic |
| 28685396 | Cross-over randomized clinical trial | 33 healthy individuals | Both (♀=51.5%) | 25-55 | Hispanic, Non-Hispanic, African American, Asian, Caucasian |
| 32475982 | Case report | 1 patient with depression | Male | 28 | Caucasian |
| 16642541 | Open and controlled clinical study | 62 patients with depression | Both (♀=67%) | 60-87 | N/A |
| 16958828 | Prospective cohort study | 100 patients with depression | Both (♀=46%) | 41-63 | N/A |
| 17803873 | Cross-sectional study | 199 patients with depression | Both | N/A | Caucasian, Asian American |
| 19822698 | Case report | 1 patient with depression | Female | 42 | N/A |
| 26406933 | In vitro preclinical study | Polymorphic variants of CYP2C19 | N/A | N/A | N/A |
| 21099743 | Cross-sectional study | 95 patients with depression | Both (♀=75.8%) | 18-81 | Non-Hispanic, Black, Hispanic |
| 24941211 | Case report | 1 patient with depression | Female | 47 | N/A |
| 29327975 | Randomized, blinded controlled clinical trial | 57 patients with depression | Both (♀=66.7%) | 23-58 | Indo- and Afro-Trinidadian |
| 25245581 | Postmortem study | 94 forensic autopsy cases | Both | 18-86 | N/A |
| 30578947 | Open, randomized, crossover clinical trials | 57 patients taking duloxetine e 82 patients taking venlafaxine | Both (♀=56.8%) | 31-55 | Caucasian |
| 28480819 | Multicenter prospective cohort study | 206 patients with depression | Both | 36-60 | Caucasian |
| 30312494 | Retrospective cohort study | 1003 patients with depression | Both | N/A | Caucasian |
| 31368838 | Comprehensive searching | N/A | N/A | N/A | N/A |
| 28520361 | Overview | N/A | N/A | N/A | N/A |
| 21366359 | Overview | N/A | N/A | N/A | N/A |
| 30789308 | Randomized, open-label, crossover, replicated, four-period, four-sequence clinical trial | 36 healthy individuals | Both (♀=47.2%) | 19-37 | Caucasian, Latino and Black |
