# Supplementary material for: Pharmacogenomic biomarkers as source of evidence of the effectiveness and safety of antidepressant therapy
Source: BMC Psychiatry. 2022 Aug 30;22:576. doi: 10.1186/s12888-022-04225-2 (PMC9425945; doi:10.1186/s12888-022-04225-2)
Supplement: Supplementary file 4 — Additional file 4: Supplementary File 4. Tables with the results related to the drugs, namely the information that was found on the SmPC and/or in the systematic literature review, and the comparison with FDA table: • Clomipramine, maprotiline, fluoxetine and citalopram (Table 1 of the Supplementary File 3). • Sertraline, fluvoxamine, escitalopram and moclobemide (Table 2 of the Supplementary File 3). • Trazodone, mirtazapine, bupropion, and venlafaxine (Table 3 of the Supplementary File 3). • Reboxetine, duloxetine, agomelatine and vortioxetine (Table 4 of the Supplementary File 3). [file 12888_2022_4225_MOESM4_ESM.docx]

**SUPPLEMENTARY FILE 4**

**Table 1 of Supplementary File 4** Pairs drug/biomarker present on records, part 1 of 4

| Records | Drug/Biomarker | | | | | | | | | | | | |
| --- | --- | --- | --- | --- | --- | --- | --- | --- | --- | --- | --- | --- | --- |
| PMID | Clomipramine N06AA04 | | | | | Maprotiline N06AA21 | | Fluoxetine N06AB03 | | | Citalopram N06AB04 | | |
|  | CYP1A2 | CYP3A4 | CYP2C19 | CYP2D6 | CYP2C9 | CYP1A2 | CYP2D6 | CYP2D6 | CYP2C9 | CYP2C19 | CYP3A4 | CYP2C19 | CYP2D6 |
| 16871470 |  | X^1^ |  | X |  |  |  |  |  |  |  |  |  |
| 20531370 |  |  | X |  |  |  |  |  |  |  |  | X |  |
| 27997040 |  |  | X | X |  |  |  |  |  |  |  |  |  |
| 19698378 |  |  |  | X |  |  |  |  |  |  |  |  |  |
| 15168101 |  |  | X | X | **●^2^** |  |  |  |  |  |  |  |  |
| 28470111 |  |  | X | X |  |  |  |  |  |  |  |  |  |
| 32433340 |  |  | X | X |  |  |  |  |  |  |  |  |  |
| 24663076 |  |  |  |  |  |  |  | X | **●** |  |  |  |  |
| 22791347 |  |  |  |  |  |  |  | X | **●** | **●** |  |  |  |
| 23799451 |  |  |  |  |  |  |  | X |  | **●** |  |  |  |
| 27289413 |  |  |  |  |  |  |  | X |  | **●** |  |  |  |
| 31664715 |  |  |  |  |  |  |  |  |  |  |  | X |  |
| 31112844 |  |  |  |  |  |  |  |  |  |  |  | X | X |
| 25974703 |  |  |  |  |  |  |  |  |  |  |  | X |  |
| 30173302 |  |  |  |  |  |  |  |  |  |  |  | X | X |
| 21192344 |  |  |  |  |  |  |  |  |  |  |  | X |  |
| 27016952 |  |  |  |  |  |  |  |  |  |  |  |  | X |
| 24257813 |  |  |  |  |  |  |  |  |  |  |  | X |  |
| 30837874 |  |  |  |  |  |  |  |  |  |  |  | X |  |
| 29712478 |  |  |  |  |  |  |  |  |  |  |  | X |  |
| 12975335 |  |  |  |  |  |  |  |  |  |  |  | X |  |
| 16855453 |  |  |  |  |  |  |  |  |  |  | X | X |  |
| 25200585 |  |  | X | X |  |  |  | X |  | X |  | X | X |
| FDA |  |  |  | X |  |  |  | X |  |  |  | X | X |
| 1 X: biomarker identified on the SmPC and systematic literature review  2 ●: biomarker identified only on systematic literature review  Biomarkers whose column does not show X or ● were identified on the SmPC but did not obtain any results through the systematic literature review | | | | | | | | | | | | | |

**Table 2 of Supplementary File 4** Pairs drug/biomarker present on records, part 2 of 4

| Records | Drug/Biomarker | | | | | | | | | | |  |
| --- | --- | --- | --- | --- | --- | --- | --- | --- | --- | --- | --- | --- |
| PMID | Sertraline N06AB06 | | | | Fluvoxamine N06AB08 | | Escitalopram N06AB10 | | | Moclobemide N06AG02 | |  |
|  | CYP3A4 | CYP2C19 | CYP2D6 | CYP2B6 | CYP2D6 | CYP2C19 | CYP3A4 | CYP2C19 | CYP2D6 | CYP2C19 | CYP2D6 |  |
| 25974703 |  | X^1^ |  |  | X |  |  | X |  |  |  |  |
| 29136336 |  | X | **●^2^** | X |  |  |  |  |  |  |  |  |
| 31649299 |  | X |  |  |  |  |  |  |  |  |  |  |
| 26830411 |  | X |  | X |  |  |  |  |  |  |  |  |
| 11452243 |  | X |  |  |  |  |  |  |  |  |  |  |
| 20547595 |  |  |  |  | X |  |  |  |  |  |  |  |
| 29988737 |  |  |  |  | X |  |  |  |  |  |  |  |
| 25200585 |  | X |  |  |  | X |  | X |  |  |  |  |
| 21926427 |  |  |  |  |  |  |  | X | X |  |  |  |
| 24302953 |  |  |  |  |  |  |  |  | X |  |  |  |
| 20350136 |  |  |  |  |  |  |  | X | X |  |  |  |
| 29325448 |  |  |  |  |  |  |  | X |  |  |  |  |
| 24014145 |  |  |  |  |  |  |  | X | X |  |  |  |
| FDA |  |  |  |  | X |  |  | X | X |  |  |  |
| 1 X: biomarker identified on the SmPC and systematic literature review  2 ●: biomarker identified only on systematic literature review  Biomarkers whose column does not show X or ● were identified on the SmPC but did not obtain any results through the systematic literature review | | | | | | | | | | | | |

**Table 3 of Supplementary File 4** Pairs drug/biomarker present on records, part 3 of 4

| Records | Drug/Biomarker | | | | | | | | | | | | | | | | | |
| --- | --- | --- | --- | --- | --- | --- | --- | --- | --- | --- | --- | --- | --- | --- | --- | --- | --- | --- |
| PMID | Trazodone N06AX05 | | Mirtazapine N06AX11 | | | | Bupropion N06AX12 | | | | | | | | Venlafaxine N06AX16 | | | |
|  | CYP3A4 | CYP2D6 | CYP1A2 | CYP3A4 | CYP2D6 | CYP2B6 | CYP1A2 | CYP2A6 | CYP3A4 | CYP2B6 | CYP2C9 | CYP2E1 | CYP2D6 | CYP2C19 | CYP3A4 | CYP2D6 | CYP2C19 | |
| 27289413 |  |  |  |  |  |  |  |  |  |  |  |  |  |  |  | X^1^ |  | |
| 25200585 |  |  |  |  |  |  |  |  |  | X |  |  |  |  |  | X |  | |
| 24014145 |  |  |  |  |  |  |  |  |  |  |  |  |  |  |  | X | **●^2^** | |
| 29061081 | X | **●** |  |  |  |  |  |  |  |  |  |  |  |  |  |  |  | |
| 9335086 |  | **●** |  |  |  |  |  |  |  |  |  |  |  |  |  |  |  | |
| 31100205 |  |  |  |  | X |  |  |  |  |  |  |  |  |  |  |  |  | |
| 26595747 |  |  | X |  | X |  |  |  |  |  |  |  |  |  |  |  |  | |
| 22926595 |  |  | X |  | X | **●** |  |  |  |  |  |  |  |  |  |  |  | |
| 14514498 |  |  |  |  | X |  |  |  |  |  |  |  |  |  |  |  |  | |
| 14515060 |  |  |  |  |  |  |  |  |  | X | X |  | **●** |  |  |  |  | |
| 26608082 |  |  |  |  |  |  |  |  |  | X |  |  |  |  |  |  |  | |
| 23344581 |  |  |  |  |  |  |  |  |  | X |  |  |  |  |  |  |  | |
| 23238783 |  |  |  |  |  |  |  |  |  | X |  |  |  |  |  |  |  | |
| 15083067 |  |  |  |  |  |  |  |  |  | X |  |  |  |  |  |  |  | |
| 28685396 |  |  |  |  |  |  |  |  |  | X |  |  |  | **●** |  |  |  | |
| 32475982 |  |  |  |  |  |  |  |  |  |  |  |  |  | **●** |  |  |  | |
| 16642541 |  |  |  |  |  |  |  |  |  |  |  |  |  |  |  | X |  | |
| 16958828 |  |  |  |  |  |  |  |  |  |  |  |  |  |  |  | X |  | |
| 17803873 |  |  |  |  |  |  |  |  |  |  |  |  |  |  |  | X |  | |
| 19822698 |  |  |  |  |  |  |  |  |  |  |  |  |  |  |  | X |  | |
| 26406933 |  |  |  |  |  |  |  |  |  |  |  |  |  |  |  | X |  | |
| 21099743 |  |  |  |  |  |  |  |  |  |  |  |  |  |  |  | X | **●** | |
| 24941211 |  |  |  |  |  |  |  |  |  |  |  |  |  |  |  | X |  | |
| 29327975 |  |  |  |  |  |  |  |  |  |  |  |  |  |  |  | X | **●** | |
| 25245581 |  |  |  |  |  |  |  |  |  |  |  |  |  |  | X | X | **●** | |
| 30578947 |  |  |  |  |  |  |  |  |  |  |  |  |  |  |  | X | **●** | |
| 28480819 |  |  |  |  |  |  |  |  |  |  |  |  |  |  |  | X | **●** | |
| 30312494 |  |  |  |  |  |  |  |  |  |  |  |  |  |  | X | X | **●** | |
| 31368838 |  |  |  |  |  |  |  |  |  |  |  |  |  |  | X | X | **●** | |
| 28520361 |  |  |  |  |  |  |  |  |  |  |  |  |  |  |  | X |  | |
| FDA |  |  |  |  |  |  |  |  |  |  |  |  | X |  |  |  |  | |
| \| 1 X: biomarker identified on the SmPC and systematic literature review  2 ●: biomarker identified only on systematic literature review  Biomarkers whose column does not show X or ● were identified on the SmPC but did not obtain any results through the systematic literature review \| \| --- \| | | | | | | | | | | | | | | | | | |  |

**Table 4 of Supplementary File 4** Pairs drug/biomarker present on records, part 4 of 4

| Records | Drug/Biomarker | | | | | | | | | |
| --- | --- | --- | --- | --- | --- | --- | --- | --- | --- | --- |
| PMID | Reboxetine N06AX18 | Duloxetine N06AX21 | | Agomelatine  N06X22 | | | Vortioxetine N06AX26 | | | |
|  | CYP3A4 | CYP1A2 | CYP2D6 | CYP1A2 | CYP2C9 | CYP2C19 | CYP3A4 | CYP3A5 | CYP2C9 | CYP2D6 |
| 25200585 |  |  |  | X^1^ |  |  |  |  |  |  |
| 21366359 |  | X | X |  |  |  |  |  |  |  |
| 30789308 |  |  |  | X | X |  |  |  |  |  |
| FDA |  |  | X |  |  |  |  |  |  | X |
| \| \| 1 X: biomarker identified on the SmPC and systematic literature review  2 ●: biomarker identified only on systematic literature review  Biomarkers whose column does not show X or ● were identified on the SmPC but did not obtain any results through the systematic literature review \| \| --- \| \| \| --- \| --- \| | | | | | | | | | | |
