# Supplementary material for: Pharmacogenomic biomarkers as source of evidence of the effectiveness and safety of antidepressant therapy
Source: BMC Psychiatry. 2022 Aug 30;22:576. doi: 10.1186/s12888-022-04225-2 (PMC9425945; doi:10.1186/s12888-022-04225-2)
Supplement: Supplementary file 5 — Additional file 5: Supplementary File 5. Levels of evidence and GRADE of the studies included in the systematic literature review. [file 12888_2022_4225_MOESM5_ESM.docx]

**SUPPLEMENTARY FILE 5**

Classification of the levels of evidence and GRADE of the studies included in the systematic literature review

| PMID | Drug | Biomarker | Type of Study | Level of Evidence | Grade of Recommendation |
| --- | --- | --- | --- | --- | --- |
| 16871470 | Clomipramine | CYP2D6  CYP3A4 | Case report | 4 | C |
| 20531370 | Clomipramine  Citalopram | CYP2C19 | Retrospective cohort study | 2B | B |
| 27997040 | Clomipramine | CYP2C19 CYP2D6 | Guideline | 5 | D |
| 19698378 | Clomipramine | CYP2D6 | Prospective cohort study | 2B | B |
| 15168101 | Clomipramine | CYP2C19 CYP2D6 CYP2C9 | Exploratory study | 4 | C |
| 28470111 | Clomipramine | CYP2C19 CYP2D6 | Case report | 4 | C |
| 32433340 | Clomipramine | CYP2C19 CYP2D6 | Prospective cohort study | 2B | B |
| 24663076 | Fluoxetine | CYP2D6 CYP2C9 | Prospective cohort study | 2B | B |
| 22791347 | Fluoxetine | CYP2D6 CYP2C9 CYP2C19 | Scoping review | 4 | C |
| 23799451 | Fluoxetine | CYP2D6 CYP2C19 | Case report | 4 | C |
| 27289413 | Fluoxetine Venlafaxine | CYP2D6 CYP2C19 | Guideline | 5 | D |
| 31664715 | Citalopram | CYP2C19 | Comment | 5 | D |
| 31112844 | Citalopram | CYP2C19 CYP2D6 | Systematic literature review | 2A | B |
| 25974703 | Citalopram Sertraline Fluvoxamine Escitalopram | CYP2C19 CYP2D6 | Guideline | 5 | D |
| 30173302 | Citalopram | CYP2C19 CYP2D6 | Case-control study | 3B | B |
| 21192344 | Citalopram | CYP2C19 | Prospective cohort study | 2B | B |
| 27016952 | Citalopram | CYP2D6 | In vitro preclinical study | 2C | B |
| 24257813 | Citalopram | CYP2C19 | Partially randomized, open-label, multicenter study | 1C | A |
| 30837874 | Citalopram | CYP2C19 | Retrospective cohort study | 2B | B |
| 29712478 | Citalopram | CYP2C19 | Editorial | 5 | D |
| 12975335 | Citalopram | CYP2C19 | Randomized controlled clinical trial | 1B | A |
| 16855453 | Citalopram | CYP3A4 CYP2C19 | Prospective cohort study | 2B | B |
| 29136336 | Sertraline | CYP2C19 CYP2D6 CYP2B6 | Randomized, open-label, crossover, two-period, two-sequence, single-center clinical trials | 1B | A |
| 31649299 | Sertraline | CYP2C19 | Retrospective cohort study | 2B | B |
| 26830411 | Sertraline | CYP2C19 CYP2B6 | Cross-sectional study | 4 | C |
| 11452243 | Sertraline | CYP2C19 | Randomized controlled clinical trial | 1B | A |
| 20547595 | Fluvoxamine | CYP2D6 | Cross-sectional study | 4 | C |
| 29988737 | Fluvoxamine | CYP2D6 | Prospective cohort study | 2B | B |
| 25200585 | Clomipramine Fluoxetine Citalopram Sertraline Fluvoxamine Escitalopram Bupropion  Venlafaxine Agomelatine | CYP2C19 CYP2D6 CYP2B6 CYP1A2 | Critical review | 4 | C |
| 21926427 | Escitalopram | CYP2C19 CYP2D6 | Prospective cohort study | 2B | B |
| 24302953 | Escitalopram | CYP2D6 | Non-randomized clinical trial | 2B | B |
| 20350136 | Escitalopram | CYP2C19 CYP2D6 | Observational case-only design study | 2C | B |
| 29325448 | Escitalopram | CYP2C19 | Retrospective cohort study | 2B | B |
| 24014145 | Escitalopram Venlafaxine | CYP2C19 CYP2D6 | Non-randomized clinical trial | 4 | C |
| 29061081 | Trazodone | CYP3A4 CYP2D6 | Randomized, open-label, crossover, two-period, two-sequence, single-center, blinded clinical trial | 1B | A |
| 9335086 | Trazodone | CYP2D6 | Cross-sectional study | 4 | C |
| 31100205 | Mirtazapine | CYP2D6 | Prospective cohort study | 2B | B |
| 26595747 | Mirtazapine | CYP1A2 CYP2D6 | Cross-sectional study | 4 | C |
| 22926595 | Mirtazapine | CYP1A2 CYP2D6 CYP2B6 | Multicenter non-randomized clinical trial | 4 | C |
| 14514498 | Mirtazapine | CYP2D6 | Double-blind, randomized clinical trial | 1B | A |
| 14515060 | Bupropion | CYP2B6 CYP2C9 CYP2D6 | Randomized controlled clinical trial | 1B | A |
| 26608082 | Bupropion | CYP2B6 | In vitro preclinical study | 5 | D |
| 23344581 | Bupropion | CYP2B6 | Randomized controlled clinical trial | 1B | A |
| 23238783 | Bupropion | CYP2B6 | Non-randomized clinical trial | 4 | C |
| 15083067 | Bupropion | CYP2B6 | In vitro preclinical study | 5 | D |
| 28685396 | Bupropion | CYP2B6 CYP2C19 | Cross-over randomized clinical trial | 1B | A |
| 32475982 | Bupropion | CYP2C19 | Case report | 4 | C |
| 16642541 | Venlafaxine | CYP2D6 | Open and controlled clinical study | 1B | A |
| 16958828 | Venlafaxine | CYP2D6 | Prospective cohort study | 2B | B |
| 17803873 | Venlafaxine | CYP2D6 | Cross-sectional study | 4 | C |
| 19822698 | Venlafaxine | CYP2D6 | Case report | 4 | C |
| 26406933 | Venlafaxine | CYP2D6 | In vitro preclinical study | 5 | D |
| 21099743 | Venlafaxine | CYP2D6 CYP2C19 | Cross-sectional study | 4 | C |
| 24941211 | Venlafaxine | CYP2D6 | Case report | 4 | C |
| 29327975 | Venlafaxine | CYP2D6 CYP2C19 | Randomized controlled clinical trial | 1B | A |
| 25245581 | Venlafaxine | CYP3A4 CYP2D6 CYP2C19 | Postmortem study | 4 | C |
| 30578947 | Venlafaxine | CYP2D6 CYP2C19 | Open, randomized, crossover clinical trials | 1B | A |
| 28480819 | Venlafaxine | CYP2D6 CYP2C19 | Multicenter prospective cohort study | 2B | B |
| 30312494 | Venlafaxine | CYP3A4 CYP2D6 CYP2C19 | Retrospective cohort study | 2B | B |
| 31368838 | Venlafaxine | CYP3A4 CYP2D6 CYP2C19 | Comprehensive searching | 4 | C |
| 28520361 | Venlafaxine | CYP2D6 | Overview | 4 | C |
| 21366359 | Duloxetine | CYP1A2 CYP2D6 | Overview | 4 | C |
| 30789308 | Agomelatine | CYP1A2 CYP2C9 | Randomized, open-label, crossover, replicated, four-period, four-sequence clinical trial | 1B | A |
